# Ganoderic acid C2 exerts the pharmacological effects against cyclophosphamide-induced immunosuppression: a study involving molecular docking and experimental validation

**DOI:** 10.1038/s41598-023-44394-y

**Published:** 2023-10-18

**Authors:** Yuchen Liu, Dongsheng Tan, Hong Cui, Jihua Wang

**Affiliations:** 1 School of Life Science and Technology, Harbin Normal University (Songbei Campus), No. 1, Shida Road, Hulan District, Harbin, 150025 Heilongjiang Province China; 2 College of Life Science, Northeast Forestry University, Harbin, 150040 China

**Keywords:** Computational biology and bioinformatics, Immunology

## Abstract

Triterpenoids, as the main active ingredient of *Ganoderma lucidum* fermented extract, exert multiple pharmacological activities, including immunomodulatory properties. Our study aimed to reveal the pharmacological effects and potential mechanisms of Ganoderic acid C2 (GAC) against cyclophosphamide (CY)-associated immunosuppression. Target genes were collected from several public databases, including the DisGeNET, Comparative Toxicogenomics Database, GeneCards, and PharmMapper. STRING database was used to construct the protein–protein interaction of network. Subsequently, molecular docking was carried out to visualize the protein-GAC interactions. Experimental validations, including ELISA and qRT-PCR were performed to confirm the pharmacological activities of GAC on CY-induced immunosuppression model. A total of 56 GAC-related targets were identified to be closely associated with CY-induced immunosuppression. Enrichment analyses results revealed that these targets were mainly involved in immune and inflammatory response-related pathways. STAT3 and TNF were identified as the core targets of GAC. Molecular docking indicated that GAC combined well with STAT3 and TNF protein. In addition, animal experiments indicated that GAC improved immunity as well as STAT3 and TNF genes expression in CY-induced immunosuppression, which further verified the prediction through bioinformatics analysis and molecular docking. We successfully revealed the potential therapeutics mechanisms underlying the effect of GAC against CY-induced immunosuppression based on the combination of bioinformatics analysis, molecular docking, and animal experiments. Our findings lay a theoretical foundation for the in-depth development and utilization of *Ganoderma lucidum* fermentation product in the future, and also provide theoretical guidance for the development of innovative drugs that assist in improving immunity.

## Introduction

The immune system is an interactive network of cytokines, humoral factors, cells, and lymphoid organs^1^. As an important defense system of the body, the immune system has important physiological functions. It protects the host from the damage of pathogenic microorganisms by identifying and monitoring non-self and self substances, so as to maintain the health of the body^2,3^. When tumor cells are killed by drugs or radiation, the body’s immune function and bone marrow hematopoietic system will also be damaged, resulting in immunosuppression^4^. Cyclophosphamide (CY) is a commonly used antineoplastic drug in clinical practice. In addition, it is also a highly effective immunosuppressant, commonly used in blood and bone marrow transplantation^5^. It could cause oxidative stress, immunosuppression, bone marrow suppression, and other side effects, which are the primary limiting factors in clinical chemotherapy^6,7^. In clinical practice, in order to reduce those side effects, chemotherapy patients usually take immunoactive substances to enhance immunity^8^. Thus, it is very important to develop novel immunomodulators that could effectively reduce CY-induced immunodeficiency.

Many natural immunomodulators, including polypeptides, flavonoids, and polysaccharides, have the ability to boost immunity and improve CY-induced immunodeficiency^9–12^. *Ganoderma lucidum* has long been used primarily for the prevention and treatment of various diseases. More and more studies have shown that it has immunomodulatory functions^13,14^. Triterpenoids and polysaccharides are the major bioactive ingredients in *Ganoderma lucidum*^15^. Triterpenoids have a wide range of biological activities, including antioxidation, hepatoprotective, anti-inflammatory, anti-apoptotic, and immune restoration effects^16–19^. Ganoderic acid C2 (GAC) is a bioactive triterpenoid in *Ganoderma lucidum* fermented extract. However, the immunomodulatory effects of GAC in CY-induced immunosuppressed mice remain unclear.

**Figure 1:**
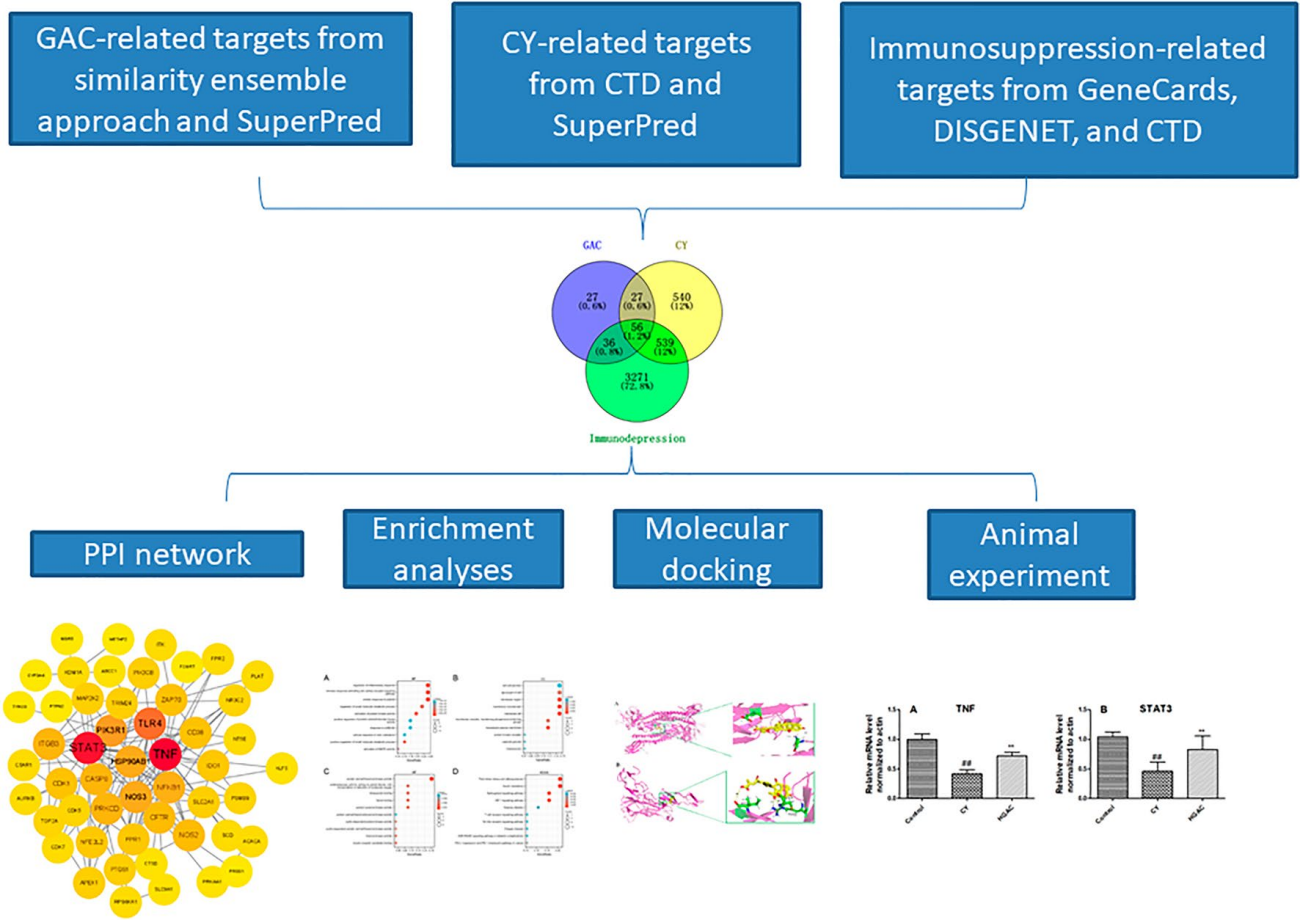
Workflow of GAC in the treatment of CY-induced immunodeficiency.

Network pharmacology is a tool for systematically describing complex interactions between drugs and biological systems from a network perspective^20^. Molecular docking, an in silico approach, is widely recognized as one of the most prevalent and effective structure-based methods for predicting the interactions between molecules and biological targets^21,22^. In the present study, a network pharmacology prediction and molecular docking-based approach, combined with animal experiments verification, was carried out to reveal the potential therapeutic mechanisms and targets of GAC against the CY-induced immunodeficiency. The workflow of the research is presented in Fig. 1.

## Materials and methods

### Collection of CY-associated targets

The CY-related targets were collected from several online databases by using “Cyclophosphamide” as keywords, including the Comparative Toxicogenomics Database (CTD, https://ctdbase.org/)^23^, and the SuperPred (https://prediction.charite.de/index.php)^24^. The Retrieve/ID mapping tool (www.uniprot.org) was used to transform protein target manes into their official gene symbols. Finally, CY-related targets were obtained by removing duplicate genes.

### Prediction of GAC-related targets

The potential targets of GAC were obtained by using following databases by entering a SMILES of GAC: the SuperPred (https://prediction.charite.de/index.php) and similarity ensemble approach (https://sea.bkslab.org/)^25^. The Retrieve/ID mapping tool (www.uniprot.org) was used to transform protein target manes into their official gene symbols. Finally, GAC-related targets were collected by removing duplicate genes.

### Screening of immunosuppression-related targets

We searched the GeneCards database (https://www.genecards.org/)^26^, the DISGENET database (https://www.disgenet.org/)^27^, and the Comparative Toxicogenomics Database (CTD, https://ctdbase.org/) to collect potential targets of immunosuppression by using “immunosuppression” or “leukopenia” as the search keywords. Finally, immunosuppression-related targets were collected by removing duplicate genes.

### The protein–protein interaction (PPI) network construction

First, the Venn diagram tool (https://bioinfogp.cnb.csic.es/tools/venny/index.html) was used to identify the intersection of GAC, CY, and immunosuppression targets. Then, those intersection targets were imported into the STRING database (https://cn.string-db.org/) to construct the PPI network; the protein interaction selection score was set to > 0.4. The TSV file was downloaded and used to visualize the PPI network results by using the Cytoscape software (version 3.2.1) (http://cytoscape.org/). The cytoHubba plugin of Cytoscape software was used to identify the core targets of GAC^28^.

### Functional enrichment analysis

We identified the potential pathways by the GO and KEGG enrichment analyses to further clarify the role of the potential anti-immunosuppression targets of GAC. The enrichment analyses were performed and results were visualized by using R packages, including enrichplot, ggplot2, and clusterProfiler. The *p* < 0.05 was considered statistically significant.

### Molecular docking

We performed molecular docking analysis to investigate the potential binding mode of GAC to a macromolecular receptor. The core genes were selected for molecular docking analysis. The 3D structures of targets protein (TNF and STAT3) were downloaded from PDB (https://www.rcsb.org) databases, and the 3D conformations of proteins with a crystal resolution < 3 Å were selected. For the preparation of receptor macromolecule: firstly, the proteins were removed from solvent and proto-ligand small molecules using PyMOL software and saved as PDB format. Then using AutoDock software, the protein was subjected to de-watering, hydrogenation, charge calculation and atom type addition operations, and finally saved as a PDBQT format file. For the preparation of small molecule ligand: firstly, the small molecule ligand was opened using AutoDock software, in which the charge was adjusted and finally saved as a PDBQT file. The AutoDockTools 1.5.6 software was used to open the receptor macromolecule and ligand PDBQT files. The size of the grid was designed to cover the binding pocket, with XYZ dimensions of 80 Å × 80 Å × 80 Å. The spacing between grid points was set to 0.6 Å. The docking process utilized the Vina force field and the Lamarckian GA (4.2) algorithm. The results of molecular docking were visualized by using the PyMOL software.

### Animals

The specific-pathogen-free Kunming mice (22–25 g and 6-week-old) were purchased from the Harbin Medical University. Mice were kept with tap water and food ad libitum, and housed in air-conditioned room (relative humidity 45–65% and temperature 20–24 °C) with a 12 h dark/light cycle. The animal experimental protocol was approved by the Ethics Committee of the Harbin Normal University.

### Acute toxicity study

The mice were divided into two groups, namely the vehicle control group and GAC control group, with 6 animals in each group. GAC was dissolved in normal saline and administered to the animals at a dose of 2000 mg/kg body weight through oral gavage. The mice were monitored for 14 days to detect any signs of toxicity or mortality. The animals' behavior was closely observed for the first 4 h, and their body weight was recorded at the beginning of the experiment, on the 7th day, and at the end of the experiment.

### Drug administration

After 7 days of acclimation, all mice were divided into five groups (n = 10 per group): control group (Control), CY-induced immunosuppression group (CY), CY group of mice received with GAC at a dose of 10 mg/kg (LGAC), CY group of mice received with GAC at a dose of 20 mg/kg (MGAC), CY group of mice received with GAC at a dose of 40 mg/kg (HGAC). CY-induced immunodeficiency mice were given intragastrically with GAC (10, 20, or 40 mg/kg body weight) for consecutive 14 days. Control group was given intragastrically with normal saline. The immunodeficiency model was induced by injecting intraperitoneally with CY (50 mg/kg/d) at day 8, 10, 12 and 14 based on the previously study^29^. Control and CY groups were given saline alone with the same operation. GAC was obtained from Shanghai Standard Technology Co Ltd (Shanghai, China). After the experiments, the mice were fasted without water overnight. Then, the mice were weighted and killed by cervical dislocation. We collected spleen and thymus tissues and weighted immediately. The immune organ index was calculated based on the following formula: organ index (mg/g) = organ weight (mg) / body weight (g).

#### Inflammatory cell counts

After the last GAC treatment, the whole blood sample was collected into EDTA-2 K tubes. After mixing 100 μL of blood sample with 900 μL of phosphate buffer solution, the resulting mixture was homogenized and centrifuged at 3000 r/min for 10 min at 4 °C. Finally, the supernatant was collected. The white blood cell (WBC), neutrophil (NEUT), and lymphocyte (LYMPH) were counted using a hematology analyzer (Siemens, ADVIA 2120i, Germany).

#### Detection of serum inflammatory cytokines and immunoglobulin

After the last GAC treatment, the whole blood sample was collected into EDTA-2 K tubes. After mixing 100 μL of blood sample with 900 μL of phosphate buffer solution, the resulting mixture was homogenized and centrifuged at 3000 r/min for 10 min at 4 °C. Finally, the supernatant was collected. The level of serum inflammatory cytokines (TNF-α, IL-12, IL-4, IFN-γ) and immunoglobulin (lgA and lgG) were measure by Enzyme Linked Immunosorbent Assay (ELISA). The blood samples were collected from orbital venous plexus after the last GAC treatment. We also measured the serum inflammatory cytokines and immunoglobulin levels by ELISA kit (Jiancheng, Nanjing) based on the manufacturer’s instruction.

#### Quantitative real-time polymerase chain reaction (qRT-PCR)

Total RNA was extracted from spleen tissues using TRIzol Reagent (Invitrogen, CA, USA) based on the manufacturer’s protocol. In addition, the RNA was measured using spectrophotometry at a wavelength of 260 nm to determine its concentration, while its purity was assessed by calculating the absorbance ratio at 260/280 nm. It is recommended that the absorbance ratios of RNA samples fall within the range of 1.8 to 2.0 for optimal purity. The purified RNA was synthesized into the cDNA using the cDNA synthesis kits (Takara Bio). Then, the qRT-PCR was performed on a StepOnePlus Real-time PCR system (Applied Biosystems, CA, USA). The 2^−ΔΔCt^ method was used to measure the mRNA expression levels. The primers were presented in Table S1.

#### Statistical analysis

The GraphPad Prism 8.0 software was used to perform the data analysis. Experimental data were shown as mean ± standard deviation (SD). One-way analysis of variance was used to compare the group differences. *p* < 0.05 was indicated statistically significant.

#### Ethics approval

All experiments protocols and animal use were approved by the Laboratory Animal Ethics Committee of Harbin Normal University.

## Results

### Screening potential targets of GAC in the treatment of CY-induced immunodeficiency

**Figure 2:**
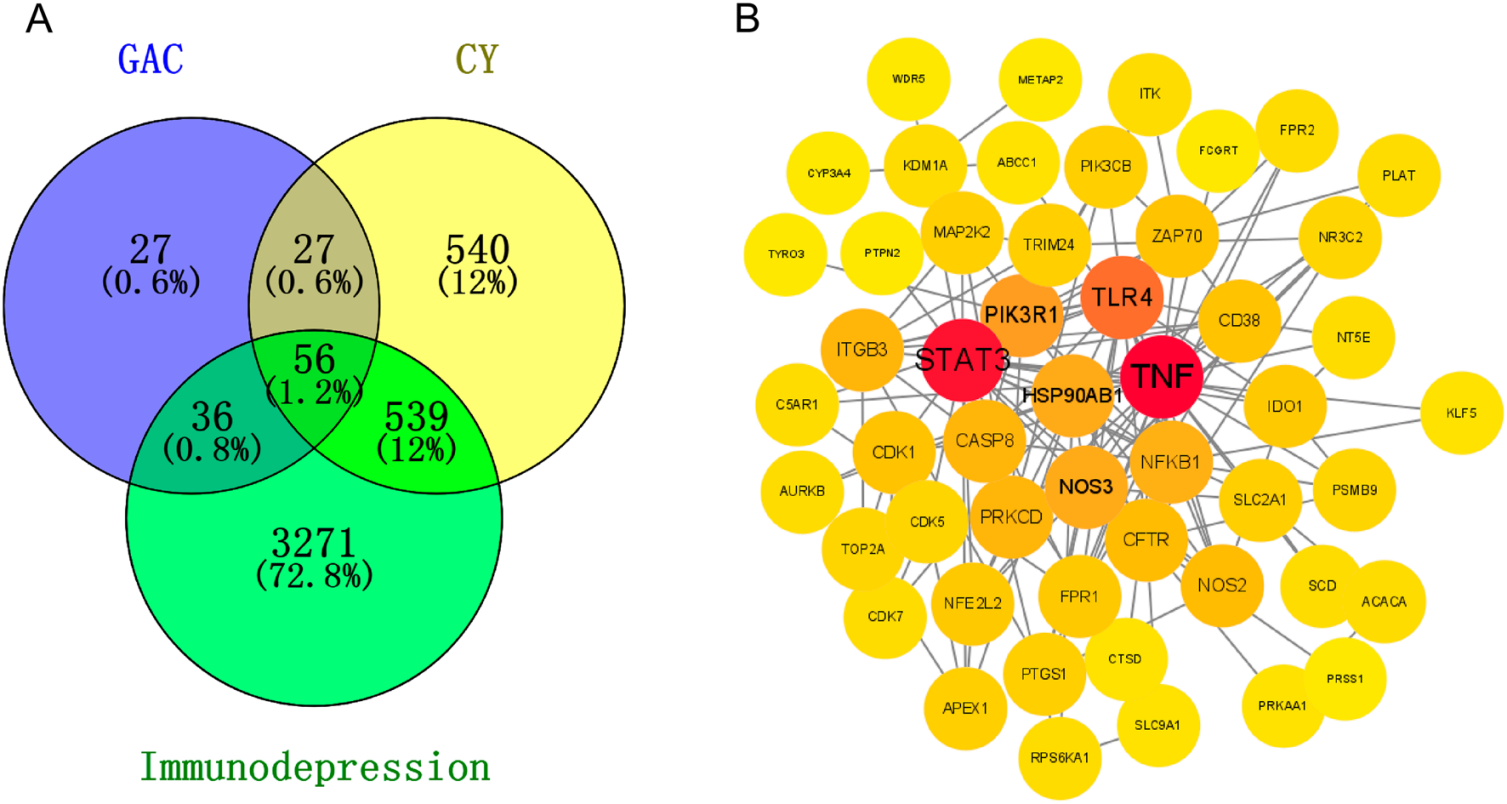
Screening potential targets of GAC in the treatment of CY-induced immunodeficiency. (**A**) The intersection of GAC-, CY- and immunodepression-related genes. (**B**) PPI network of 56 intersection targets.

In Fig. 2A, we utilized the Comparative Toxicogenomics Database and SuperPred to collect a total of 1162 targets for CY. From the SuperPred and similarity ensemble approach databases, we obtained 146 GAC-related targets. Additionally, 3902 targets associated with immunodeficiency were gathered from the GeneCards, DISGENET, and CTD databases. As a result, we identified a total of 56 intersecting genes, which were considered potential targets of GAC in treating CY-induced immunodeficiency. To visualize the interactions among these overlapping genes, we employed Cytoscape software to construct a PPI network. As depicted in Fig. 2B, the PPI network consisted of 51 nodes and 145 edges. Notably, from the PPI network, we observed that TNF, STAT3, TLR4, PIK3R1, and HSP90AB1 were significant targets for GAC intervention in CY-induced immunodeficiency.

### GO and KEGG enrichment analyses

**Figure 3:**
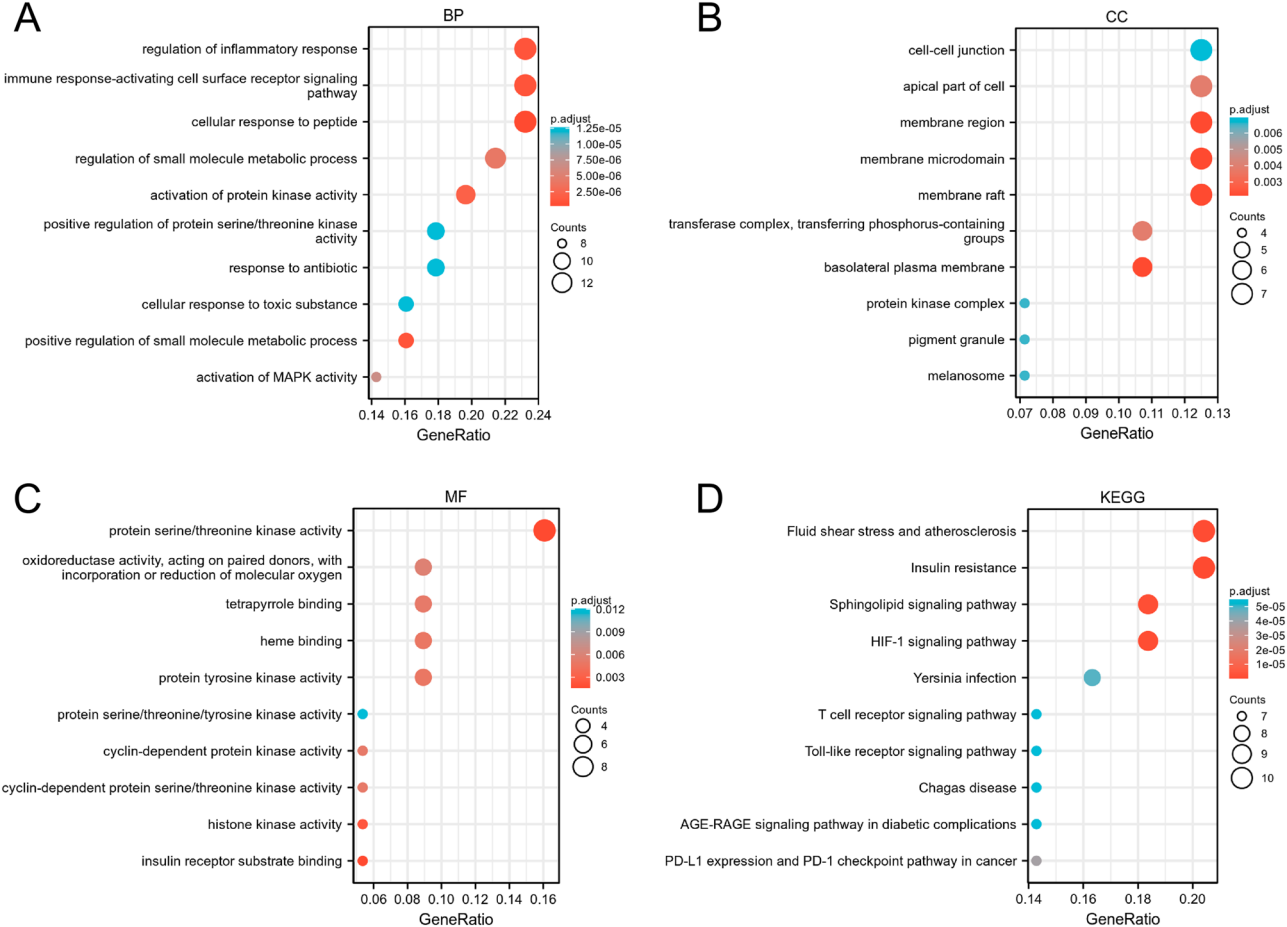
Functional enrichment analysis of 56 intersection targets. Bubble diagrams depicted the top 10 BP (**A**), CC (**B**), MF (**C**) terms and top 10 KEGG pathways.

**Figure 4:**
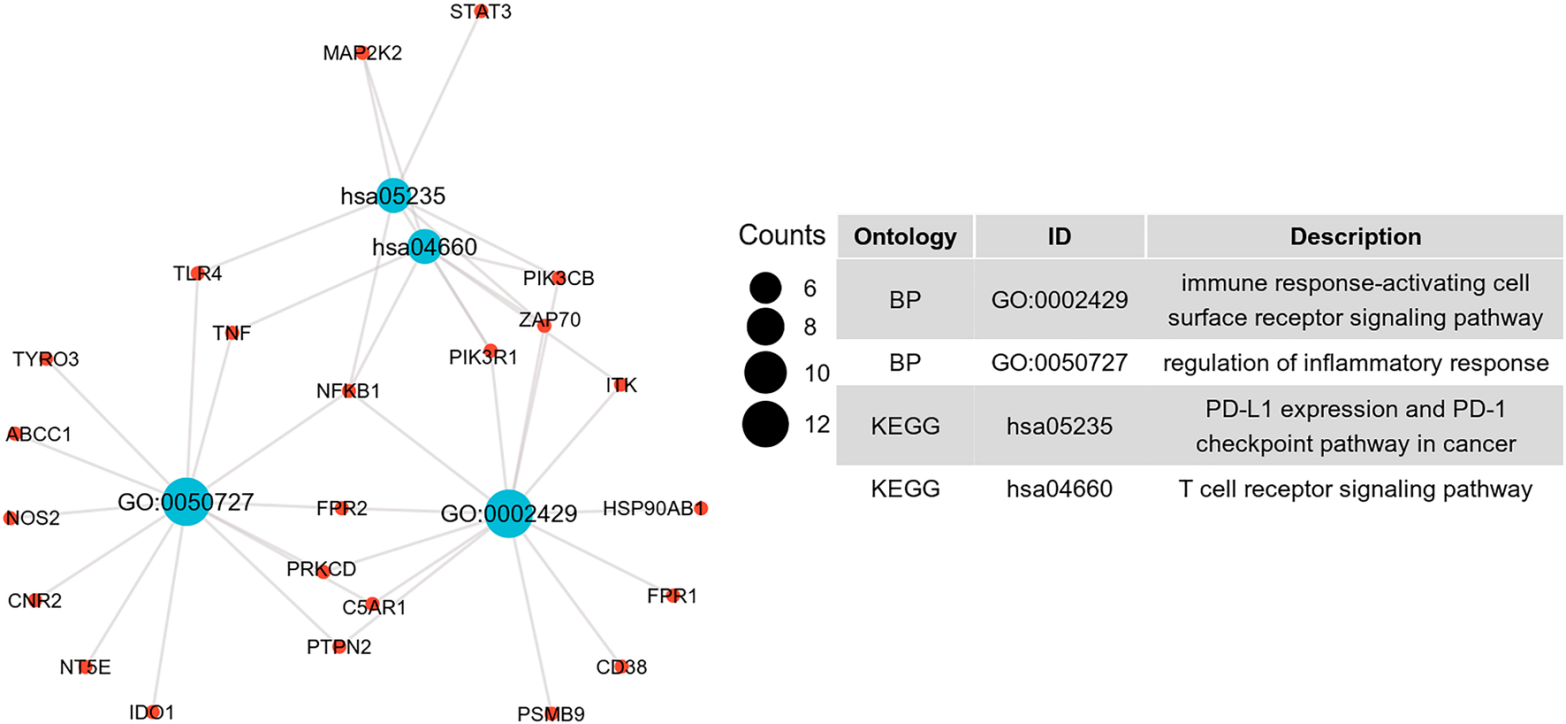
The immune-related pathways of GAC intervention CY-induced immunodeficiency. The red circular nodes represent target genes, the blue circular nodes represent immune-related pathways.

In our study, we conducted functional enrichment analyses to gain further insights into the biological functions of the potential genes targeted by GAC in the treatment of CY-induced immunodeficiency. In the category of biological processes (BP), the target genes exhibited significant enrichment in various pathways, including regulation of inflammatory response, immune response-activating cell surface receptor signaling pathway, cellular response to peptide, regulation of small molecule metabolic process, and activation of protein kinase activity (Fig. 3A). Regarding the cellular component (CC) category, the target genes were primarily associated with cell–cell junction, the apical part of the cell, membrane region, membrane microdomain, and membrane raft (Fig. 3B). In the molecular function (MF) category, the target genes were mainly involved in protein serine/threonine kinase activity, tetrapyrrole binding, heme binding, protein tyrosine kinase activity, and cyclin-dependent protein kinase activity (Fig. 3C). In the KEGG category, the target genes were mainly involved in fluid shear stress and atherosclerosis, insulin resistance, sphingolipid signaling pathway, HIF-1 signaling pathway, yersinia infection, T cell receptor signaling pathway, Toll-like receptor signaling pathway, PD-L1 expression and PD-1 checkpoint pathway in cancer, etc. (Fig. 3D). Notably, the potential mechanisms underlying the therapeutic effects of GAC against CY-induced immunosuppression involve various immune-related pathways. These pathways include the regulation of inflammatory response, immune response-activating cell surface receptor signaling pathway, PD-L1 expression and PD-1 checkpoint pathway in cancer, and T cell receptor signaling pathway (Fig. 4).

### Core targets identification

**Figure 5:**
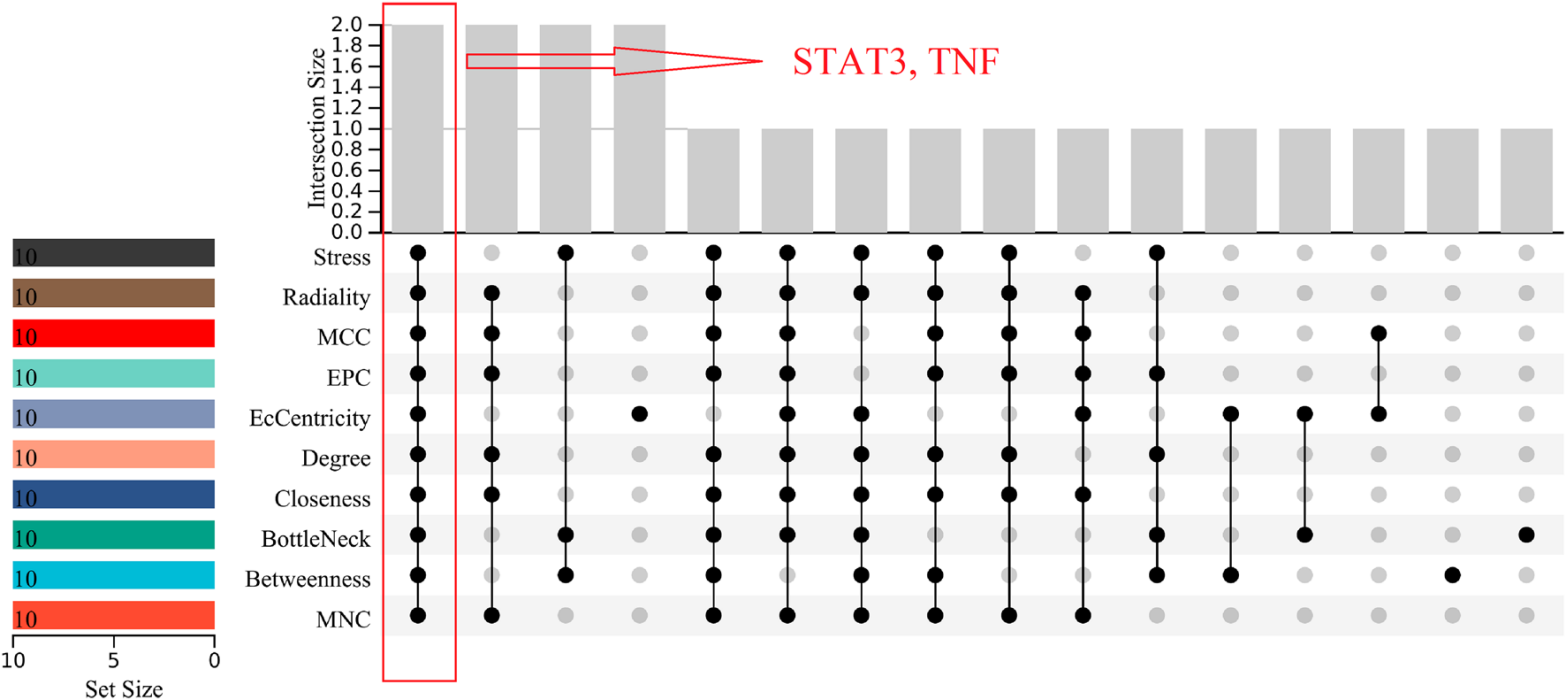
Core targets identification. Ten algorithms (stress, radiality, MCC, EPC, EcCentricity, degree, closeness, bottleneck, betweenness, and MNC) were used to identify hub genes based on R package “UpSet”.

Based on the results shown in Table 1, the top 10 genes were identified using 10 different topological analysis algorithms in cytoHubba. Subsequently, we employed the R package "UpSet" to determine the two core genes, namely STAT3 and TNF (Fig. 5).

**Table 1:** Top 10 genes by 10 ranked methods in cytoHubba.

Rank methods in cytoHubba									
MNC	Betweenness	BottleNeck	Closeness	Degree	EcCentricity	EPC	MCC	Radiality	Stress
Top 10 genes									
PRKCD	KDM1A	KDM1A	PRKCD	PRKCD	ZAP70	PRKCD	PRKCD	PRKCD	KDM1A
PIK3R1	SLC2A1	PIK3R1	PIK3R1	PIK3R1	SLC2A1	PIK3R1	PIK3R1	PIK3R1	PIK3R1
NOS3	PIK3R1	NOS3	NOS3	NOS3	NOS3	NOS3	NOS3	NOS3	NOS3
HSP90AB1	HSP90AB1	HSP90AB1	HSP90AB1	HSP90AB1	HSP90AB1	ITGB3	NFE2L2	HSP90AB1	HSP90AB1
TLR4	ITGB3	ITGB3	TLR4	ITGB3	FPR1	TLR4	TLR4	TLR4	ITGB3
CASP8	TLR4	RPS6KA1	CASP8	TLR4	NFE2L2	CASP8	CASP8	CASP8	TLR4
STAT3	ABCC1	STAT3	STAT3	CASP8	TNF	STAT3	STAT3	STAT3	STAT3
NFKB1	STAT3	TNF	NFKB1	STAT3	STAT3	NFKB1	NFKB1	NFKB1	TNF
TNF	TNF	IDO1	TNF	NFKB1	IDO1	TNF	TNF	TNF	NFKB1
NOS2	CFTR	CFTR	NOS2	TNF	NOS2	NOS2	NOS2	NOS2	CFTR

### Molecular docking

**Figure 6:**
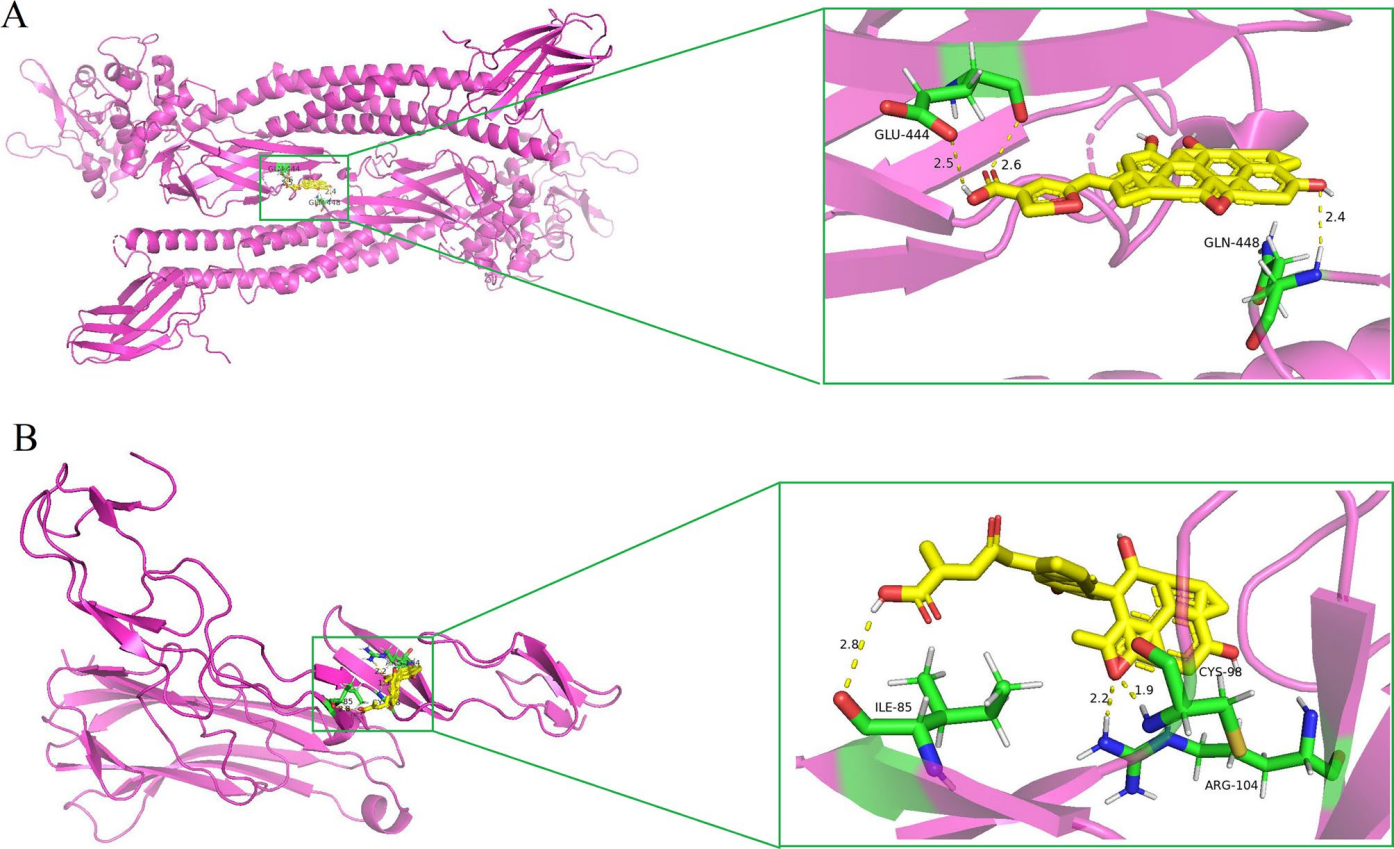
Molecular docking analysis of GAC binding to two hub targets. Protein (**A**) STST3 (6TLC), (**B**) TNF (1TNR) were exhibited interacting with the GAC small molecular. The yellow stick model represents GAC. The light dashed line represents hydrogen bond. The green stick model represents residue in the binding sites. The pink represents large molecular protein.

In this study, we conducted molecular docking analysis on GAC using two hub proteins, STAT3 and TNF, to further investigate the potential targets of GAC against CY-induced immunosuppression. The key binding sites between GAC and the amino acid residues of the target proteins were illustrated in Fig. 6, demonstrating a strong binding affinity between GAC and the target proteins (STAT3 and TNF). The binding energy between GAC and STAT3 was calculated to be − 12.2 kcal/mol, while for TNF it was − 9.29 kcal/mol. These results from the molecular docking analysis were consistent with the findings from network pharmacology, confirming the accuracy of the predicted results obtained through network pharmacology.

### Acute toxicity study

There were no observed alterations in behavioral parameters in mice administered with a dose of 2000 mg/kg of GAC orally. Additionally, there were no significant changes in body weight recorded throughout the duration of the study (Table S2). Moreover, the mice administered a dose of 2000 mg/kg of GAC exhibited no signs of toxicity and survived, suggesting that a single dose of GAC at this concentration is well-tolerated by mice.

### Effect of GAC on body weight and immune organ index in CY-induced immunodeficiency

**Figure 7:**
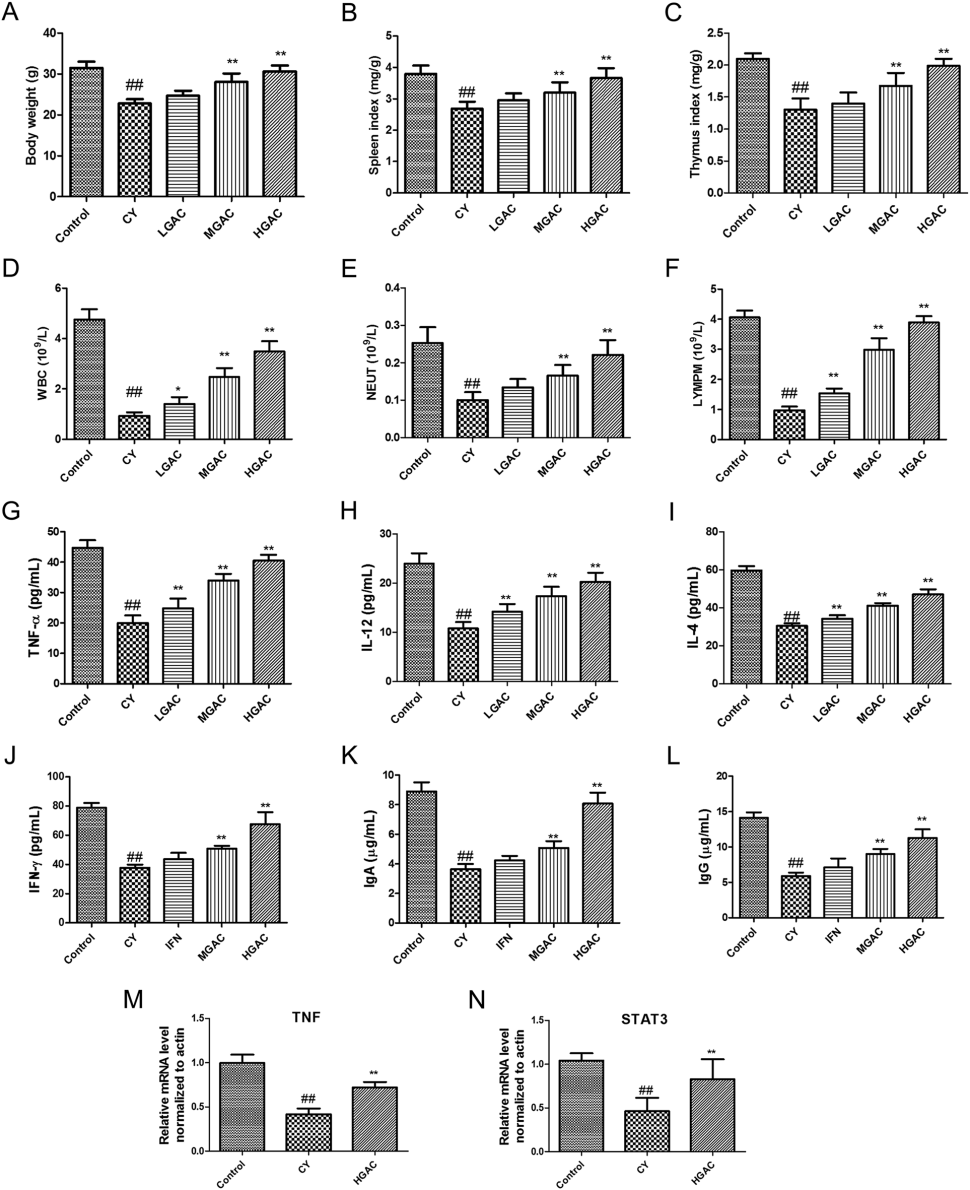
GAC exhibits a protective effect on mice with immunosuppression induced by CY. Effect of GAC on body weight (**A**), spleen index (**B**), thymus index (**C**), WBC (**D**), NEUT (**E**), and LYMPM (**F**) in CY-treated mice. Effect of GAC on the level of TNF-α (**G**), IL-12 (**H**), IL-4 (**I**), IFN-γ (**J**), lgA (**K**) and lgG (**L**) in CY-treated mice. Effect of GAC on the mRNA expression of TNF (**M**) and STAT3 (**N**) in CY-treated mice. Data were shown as mean ± standard deviation (SD). ##*p* < 0.01, compared with the control group; *p < 0.05, compared with the CY group; **p < 0.01, compared with the CY group.

Figure 7A illustrates that the bodyweight of mice in the CY group decreased significantly compared to the control group (*p* < 0.05). However, after treatment with GAC, the mice in the MGAC and HGAC groups exhibited a significant increase in body weight when compared to the CY group (*p* < 0.05). Furthermore, the spleen and thymus indices of mice in the CY group decreased significantly compared to the control group (*p* < 0.05), as shown in Fig. 7B,C. However, after a 14-day treatment with GAC (20 or 40 mg/kg), the reduction in immune organ indices induced by CY was alleviated (*p* < 0.05).

### Effects of GAC on inflammatory cell counts in CY-induced immunodeficiency

According to the data depicted in Fig. 7D–F, the administration of CY resulted in a significant decrease in the counts of WBC, NEUT, and LYMPH in mice (*p* < 0.05). However, after a 14-day treatment with GAC (20 or 40 mg/kg), the decline in inflammatory cell counts induced by CY was significantly improved (*p* < 0.05). These results demonstrate the immunomodulatory activity of GAC in CY-treated mice.

### Effects of GAC on the level of inflammatory cytokines in CY-treated mice

According to the data illustrated in Fig. 7G–J, the administration of CY resulted in a significant decrease in the levels of inflammatory cytokines (TNF-α, IL-12, IL-4, IFN-γ) in mice (*p* < 0.05). However, after a 14-day treatment with GAC (20 or 40 mg/kg), the decline in inflammatory cytokine levels induced by CY was significantly alleviated (*p* < 0.05).

### GAC treatment promoted the generation of immunoglobulin in CY-induced immunodeficiency

According to the findings presented in Fig. 7K,L, the administration of CY led to a significant decrease in the levels of immunoglobulins (IgA and IgG) in mice (*p* < 0.05). However, after a 14-day treatment with GAC (20 or 40 mg/kg), the decline in immunoglobulin levels induced by CY was effectively reversed (*p* < 0.05).

#### GAC up-regulated core genes expression levels in CY-induced immunodeficiency

To confirm the findings of the molecular docking analysis, a qRT-PCR analysis was conducted. As depicted in Fig. 7M,N, the expressions of TNF and STAT3 were markedly up-regulated in the CY-induced immunodeficiency after a 14-day treatment with GAC (20 or 40 mg/kg) (*p* < 0.01).

## Discussion

The immunosuppressive environment plays a critical role in facilitating the development and advancement of tumors. In clinical practice, certain chemotherapeutic drugs, like cyclophosphamide (CY), not only target cancer cells, but also hinder the immune system of patients, ultimately leading to a lower rate of tumor recovery^5^. Simultaneously, prior research has indicated that prolonged chemotherapy can result in the depletion of T cells and lymphopenia^30^. As a result, the discovery of new medications that can safeguard against the immunosuppressive impact of CY is of utmost importance. Chinese herbal medicine and natural products offer a complementary and alternative approach to combating CY-induced immunodeficiency due to their ability to target multiple pathways and mechanisms^31–33^. *Ganoderma lucidum* has been utilized for a considerable period to prevent and treat a diverse range of diseases owing to its immunomodulatory properties^13,14^. Ganoderic acid C2 (GAC) is a significant bioactive component found in *Ganoderma lucidum*. However, the therapeutic effects and mechanisms of GAC in treating immunodeficiency remain unexplored. Network pharmacology, as an advanced technology, is primarily employed to predict the intricate interaction between drugs and diseases. It has proven useful in researching novel drug targets, understanding drug mechanisms, and discovering new medications^34,35^. In our study, we integrated network pharmacology, molecular docking, and animal experiments to investigate the potential therapeutic targets and mechanisms of GAC in combating cyclophosphamide-induced immunosuppression.

Initially, we identified 56 target genes that serve as therapeutic targets for GAC in combating cyclophosphamide-induced immunosuppression. Notably, two hub genes (TNF and STAT3) were identified as playing a pivotal role in the PPI network. Moreover, molecular docking results revealed that hydrogen bonding is the primary mode of interaction between target proteins and GAC, providing further validation to the network pharmacology findings. Tumor necrosis factor (TNF), an inflammatory cytokine, plays a crucial role in disease pathogenesis and maintaining homeostasis^36^. The TNF family members and their receptors are involved in activating, differentiating, proliferating, or promoting the survival of immune cells^37^. Furthermore, recent research has shown a decrease in TNF levels in mice with CY-induced immunosuppression^31,38^. Signal transducer and activator of transcription 3 (STAT3), a transcriptional regulator, plays a vital role in mature tissue function and vertebrate development including control of immunity and inflammation^39,40^. STAT3 involved in tumor immunity and inflammation through contributing to pro-oncogenic inflammatory pathways, such as IL-6-GP130-JAK and NF-kappaB pathways^41^. Moreover, recent studies showed that the JAK2/STAT3 signaling pathway was inactivated in CY-induced hematopoietic dysfunction^42–44^. In this study, it was observed that GAC could increase the expression levels of TNF and STAT3 in mice with CY-induced immunodeficiency. This suggests that the TNF and STAT3 genes are likely to be crucial in the development of CY-induced immunodeficiency, and targeting them could be a potential therapeutic approach for GAC in treating immunodeficiency.

Neutrophils, lymphocytes, and white blood cells, along with immune organs such as the thymus and spleen, as well as immunologic active materials like interleukins, interferon, and immunoglobulin, are vital components of the immune system^45,46^. Previous research has shown that natural compounds have immunomodulatory effects by activating immune cells and increasing levels of inflammatory cytokines and immunoglobulins^9,11^. In our current study, we discovered that GAC can enhance immune response in mice treated with CY, providing further confirmation of the network pharmacology findings.

## Conclusion

By utilizing network pharmacology, molecular docking, and experimental validation, our study demonstrates that GAC possesses immunomodulatory properties by activating TNF and STAT3 expression. This research provides a comprehensive understanding of the therapeutic targets and mechanisms of GAC in the treatment of immunodeficiency, integrating network pharmacology with experimental validation.

### Supplementary Information

Supplementary Tables.

## Data Availability

The data used in our study are available from the corresponding authors upon request.
