# Supplementary material for: Ganoderic acid C2 exerts the pharmacological effects against cyclophosphamide-induced immunosuppression: a study involving molecular docking and experimental validation
Source: Sci Rep. 2023 Oct 18;13:17745. doi: 10.1038/s41598-023-44394-y (PMC10584852; doi:10.1038/s41598-023-44394-y)
Supplement: Supplementary file 1 — Supplementary Tables. [file 41598_2023_44394_MOESM1_ESM.docx]

Table S1 Sequences of primers used quantitative real-time PCR.

| Gene | Forward primer (5' to 3') | Reverse primer (5' to 3') |
| --- | --- | --- |
| TNF | ATCCGCGACGTGGAACTG | ACCGCCTGGAGTTCTGGAA |
| STAT3 | CCCAAGCTTATGGCCCAATGGAATCAGCT | CCGCTCGAGTCACATGGG GGAGGTAGCGC |
| β-actin | CTTCCTGGGCATGGAGTCCT | GGAGCAATGATCTTGATCTT |

Table S2 Alteration in body weight during acute toxicity in mice.

| Day 0 | Body weight (g) | |
| --- | --- | --- |
| Animal no | Control | 2000 mg/kg |
| 1 | 30.15 | 29.18 |
| 2 | 29.61 | 28.91 |
| 3 | 28.9 | 30.15 |
| 4 | 30.27 | 31.1 |
| 5 | 30.11 | 30.18 |
| 6 | 27.51 | 27.6 |
| Day 7 | Body weight (g) | |
| Animal no | Control | 2000 mg/kg |
| 1 | 28.5 | 28.67 |
| 2 | 27.68 | 30.54 |
| 3 | 30.1 | 30.4 |
| 4 | 29.48 | 28.47 |
| 5 | 30.24 | 30.92 |
| 6 | 29.63 | 27.58 |
| Day 14 | Body weight (g) | |
| Animal no | Control | 2000 mg/kg |
| 1 | 29.15 | 28.43 |
| 2 | 28.53 | 29.4 |
| 3 | 27.19 | 28.27 |
| 4 | 31.1 | 31.6 |
| 5 | 30.57 | 30.9 |
| 6 | 32.07 | 31.5 |
